# Spondylodiscitis in a Patient Undergoing Chronic Peritoneal Dialysis Presenting to a Chiropractor: Case Report and a Review of the Literature

**DOI:** 10.7759/cureus.44312

**Published:** 2023-08-29

**Authors:** Eric Chun-Pu Chu, Kristy Yau, Steve Ming Hei Yun

**Affiliations:** 1 Chiropractic and Physiotherapy Center, New York Medical Group, Hong Kong, CHN

**Keywords:** dialysis, end-stage renal diseas, hemodialysis, chiropractor, spinal infection, peritoneal dialysis, chiropractic, spondylodiscitis

## Abstract

Spondylodiscitis is a rare but severe condition characterized by spinal and paraspinal infections that will present to healthcare providers, including chiropractors, because of neck pain. Patients who undergo peritoneal dialysis for end-stage renal disease or have a complex medical history may present with musculoskeletal complaints with complicated and uncommon underlying causes. This report reviews the possible relationship between spinal infection and dialysis, as well as the role of the chiropractor in identifying and managing critical conditions.

A 66-year-old Asian man undergoing peritoneal dialysis presented to a chiropractic clinic with pain in the left arm. The patient did not present with a fever. Upon examination, the upper limb muscle strength and reflexes were diminished. Neck pain was also experienced during the range of motion examination, and movement was limited in all directions. Magnetic resonance imaging was ordered. The patient was diagnosed with acute spondylodiscitis.

Due to its nonspecific presentation, patients with severe and urgent cases, such as spondylodiscitis, may present to chiropractic offices to seek conservative treatment of pain symptoms. This report highlights the potential role of chiropractors in the early detection and management of complex conditions such as spondylodiscitis in patients on chronic dialysis.

## Introduction

Spondylodiscitis, also known as vertebral osteomyelitis or disc space infection, is a rare but serious condition involving infection and inflammation of the intervertebral disc space and adjacent vertebrae [1]. Common pathogens that cause spondylodiscitis include *Staphylococcus aureus* and *Escherichia coli* [1]. Spondylodiscitis is more common in older patients and in patients with diabetes mellitus, immunosuppression, and a history of infection [2]. Spondylodiscitis has been reported in three per 7221 people (0.04%) who visit Hong Kong chiropractors with new episodes of lower back pain [3]. This condition is characterized by severe neck and back pain coupled with systemic symptoms such as fever, malaise, general weakness, and weight loss [4]. If left untreated, it can lead to significant morbidity, including neurological deficits and sepsis. Therefore, early diagnosis and appropriate treatment are paramount [2].

Peritoneal dialysis is a common therapeutic method for assisting the elimination of waste products and excess fluids in patients with end-stage renal disease. Patients may need to undergo peritoneal dialysis several times a day, which may be performed overnight. Usually, patients can perform the procedure independently at home with a catheter inserted into their abdomen prior to surgery [5]. Owing to its flexibility, the utilization rate of peritoneal dialysis in Hong Kong is the highest compared to that in other regions of the world [5]. Peritoneal dialysis for end-stage renal disease raises concerns about the increased risk of infections, such as peritonitis, catheter exit-site infection, and tunnel infection [6,7]. *Staphylococcus aureus* and *Pseudomonas aeruginosa* are the most common bacteria that cause infections [7]. Therefore, pyogenic infection is a potential complication in patients on dialysis who present to primary care providers for reasons other than renal disorders.

This report describes a patient with spondylodiscitis who presented to a chiropractor in Hong Kong. The patient underwent chronic peritoneal dialysis. A brief review of the literature was conducted to define a possible relationship between spinal infection and dialysis, as well as the role of chiropractors in identifying and managing critical conditions, as in this case.

## Case presentation

A 66-year-old non-smoking Asian man on peritoneal dialysis with a catheter in his abdomen presented to a chiropractic clinic with left arm pain without fever. He described the pain as sharp and intense, rating it as seven to eight out of 10 on a pain scale. The pain he had been experiencing for the last three months had an insidious onset. Furthermore, the patient reported a 10-year history of chronic neck soreness. Neither the arm nor neck pain was accompanied by numbness or tingling.

**Figure 1:**
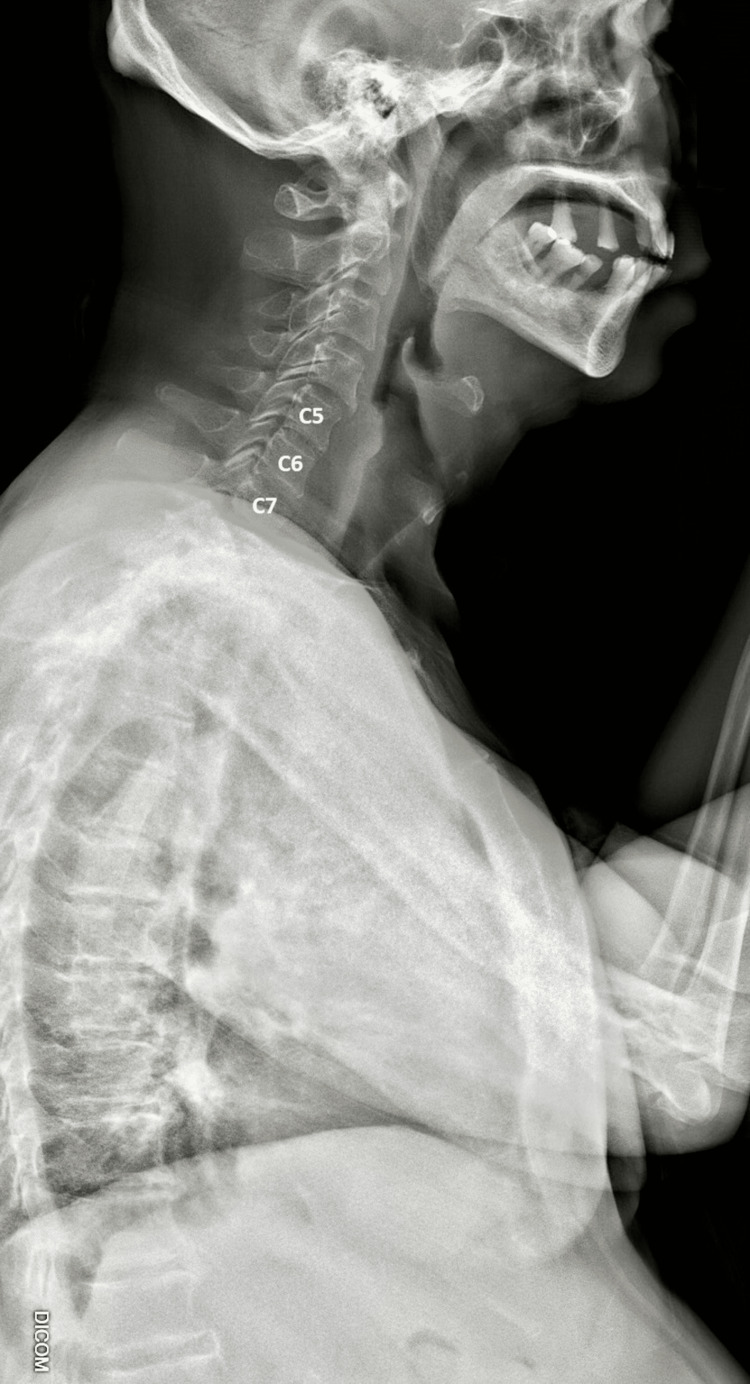
Lateral spinal radiograph Spondylosis is evident from the marginal anterior osteophytes seen in the cervical, thoracic, and lumbar spine. The disc height is mildly reduced at the C5/6 and C6/7 levels.

Upon reviewing his past medical records, it was found that the patient had been investigated with a radiograph at a public hospital approximately one year prior. Radiography did not reveal any significant findings (Figure 1). The patient also reported long-standing numbness in both toes. Interestingly, despite these musculoskeletal symptoms, the patient denied experiencing lower back pain or radiating pain.

The patient was diagnosed with kidney failure 15 years prior. He is currently undergoing peritoneal dialysis three times per day. An attempt to resolve his kidney problems was made through a kidney transplant 10 years prior. Unfortunately, the transplanted organ failed eight years after surgery. The patient was then forced to resume dialysis.

Before the development of kidney problems, the patient was actively working as a salesperson. However, because of the frequency of his dialysis treatments, he remained homebound for a considerable period. During the physical examination, the patient's range of motion was restricted in all directions in the cervical spine. This restriction intensified the pain in the arm of the patient upon flexion, and he also reported neck pain. The range of motion of the lumbar spine was within normal limits, and the patient did not experience any pain in this area.

During the physical examination, the Valsalva's maneuver resulted in increased arm pain, indicating a positive result. However, cervical compression, Jackson compression, cervical distraction, the Kemp test, slump test, toe walk, and heel walk tests yielded negative results. A neurological examination of the upper extremity revealed a reduced muscle strength of three out of five in the left biceps. The remaining results of the examination were within normal limits, with normal sensation and reflexes graded two out of five. Lower extremity muscle strength was normal at five out of five, with normal sensation and reflexes graded two out of five.

**Figure 2:**
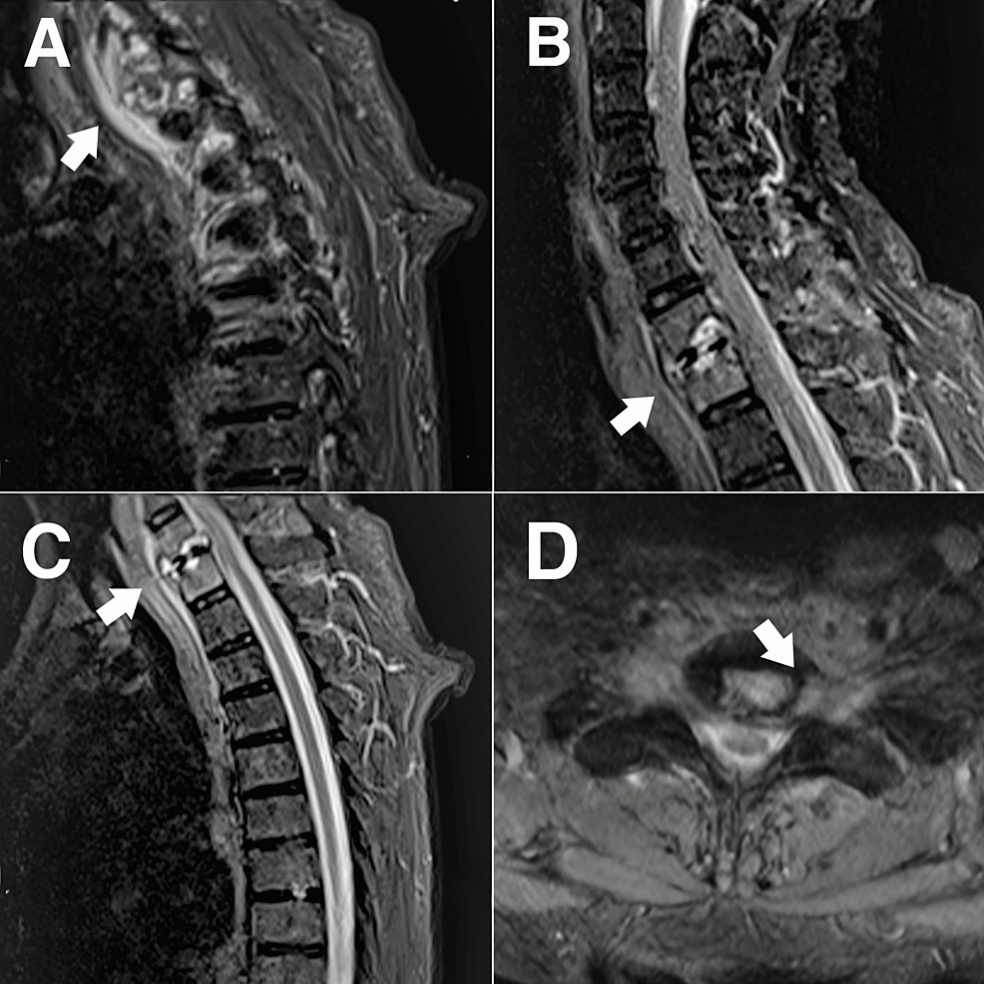
Sagittal T2-weighted magnetic resonance image (MRI) (A) Increased paravertebral soft tissue thickening/fluid collection is noted from C6/7 to T3/4 levels. (B) Increased T2-weighted hyperintensity at C7/T1 intervertebral disc with disc height loss and irregular C7/T1 endplate at the cervical scan. (C) Increased T2-weighted hyperintense signal at C7 and T1 vertebral bodies at the thoracic scan. (D) Increased paravertebral fluid collection at the C7/T1 level in the coronal view.

Considering the patient's symptoms, including advanced age and history of chronic dialysis, the chiropractor considered a differential diagnosis, including spondylodiscitis, compression fracture, malignancy, and disc herniation. The chiropractor ordered an urgent full spinal MRI scan to better evaluate any possible spinal infection, which was performed on the same day of presentation to the hospital. The MRI scan revealed an increase in T2W hyperintensity at the C7/T1 intervertebral disc, with a loss of disc height and an irregular C7/T1 endplate. An increased thickening of the paravertebral soft tissue and fluid collection were observed at levels C6/7 to T3/4. An increased hyperintense signal was also observed at the C7 and T1 vertebral bodies (Figure 2). The radiologist alerted the chiropractor regarding the urgency of these findings. The chiropractor immediately referred the patient to the emergency department.

## Discussion

Patients undergoing long-term dialysis are particularly susceptible to bacterial infections, including spondylodiscitis. First, these patients often have impaired innate and adaptive immune responses due to uremia, compromising their ability to fight infections effectively [8]. Second, repeated vascular access necessary for hemodialysis and repeated introduction of dialysate during peritoneal dialysis provide a potential entry point for pathogens and increase the risk of bacterial infection [9,10]. Furthermore, skin, soft tissue, postoperative wounds, pre-existing or synchronous genitourinary tract infection, and intra-abdominal infection are considered predisposing factors to pyogenic spinal and paraspinal infection [10,11]. Furthermore, the prevalence of comorbid conditions such as diabetes in this population of patients further increases susceptibility to infections [12]. Given these factors, spondylodiscitis, although relatively rare, can occur in patients on chronic dialysis and may present a significant diagnostic and therapeutic challenge owing to its nonspecific clinical presentation and complex medical conditions [13].

A literature search was conducted on July 23, 2023, to identify comparable cases of patients with an undiagnosed spinal infection who presented to a chiropractor. The search, which was limited to fully described case histories and excluded gray literature, used databases such as PubMed, Google Scholar, and the Index to Chiropractic Literature. The search terms included variations of the terms "chiropractor", "spondylodiscitis", "dialysis", "peritoneal dialysis", "spinal infection", and "vertebral osteomyelitis". We also performed citation tracking for the included articles.

We identified 10 relevant cases (Table 1) [3]. Eleven patients were included in these studies. The average age was 55 years. The duration of the symptoms varied greatly, with a mean duration of seven weeks. Most of the cases consisted of male patients, representing more than 90 % of the total. The most frequently reported symptom was lower back pain, which was experienced by more than 60 % of patients. All patients who provided information on pain severity (n=7) described it as severe, with a score of seven or greater on a scale of 1-10. None of the patients who reported the patient's temperature (n=7) presented a fever during their visit to the chiropractor's offices, although some patients reported experiencing subjective fever recently. The presence of indicative symptomatology and/or risk factors varied, with some patients having diabetes (n=2), night pain (n=1), bilateral radiculopathy (n=1), chronic dialysis (n=1), human immunodeficiency virus infection (n=1), or were active smokers (n=1).

**Table 1:** List of cases of spondylodiscitis presented to the chiropractor F - female; M - male; LBP - lower back pain; NR - not reported

Author, year	Patient age, sex	Symptoms and duration	Indicative symptomatology or risk factors	Fever on presentation	Diagnosis	Management
Chu, 2022 [3]	50, M	LBP, 1 week	Night pain	NR	Psoas abscess, spondylodiscitis	Pharmacotherapy
Chu, 2022 [3]	60, M	LBP with lower extremity symptoms, 1 week	Bilateral radiculopathy	NR	Epidural abscess	Pharmacotherapy
Chu, 2022 [3]	60, F	LBP and hip pain, 1 week	None	NR	Vertebral osteomyelitis	Pharmacotherapy
Chu, 2022 [14]	60, M	Severe LBP with bilateral radiation, neck pain, 2 weeks	Poorly controlled diabetes	No	Paraspinal, psoas, and epidural abscesses (pyogenic)	Intravenous antibiotics
Chu, 2023 [15]	80, M	Severe LBP with radiation to the flank, difficulty walking, 1 month	Age >70, worsening despite treatment, recent fever	No	Pyogenic spondylodiscitis, psoas abscesses, epidural abscess/phlegmon	Intravenous antibiotics
Current case	66, M	Left arm pain, severity rated 7-8/10, 3 months	Chronic peritoneal dialysis, history of kidney failure, and kidney transplant	No	Spondylodiscitis	Referral to hospital was made
Cupler, 2017 [16]	59, M	Severe thoracic spine pain, 8 days	Smoking	No	Pyogenic spondylodiscitis, epidural abscess, & phlegmon	Laminectomy of T7-8, fusion of T6-9, and antibiotics
Fogeltanz, 2006 [17]	44, M	Severe neck pain, headaches, 2 weeks	Night pain, constant pain	No	Retropharyngeal abscess	C1/2 fusion, antibiotics
Kanga, 2015 [18]	32, M	Severe LBP with radiation to thigh, 11 months	HIV	NR	Tuberculous spondylodiscitis	Pharmacotherapy
Kim, 2004 [19]	50, M	LBP with radiation to the leg, 6 weeks	None	No	Pyogenic spondylodiscitis	Intravenous antibiotics
Murphy, 2006 [20]	52, M	Severe neck pain, 1 week	Recent fever, diabetes mellitus	No	Epidural abscess	Patient passed away

As lower back pain, neck pain, and pain in the extremities are the three main reasons for seeking chiropractic care [21], chiropractors should be aware that the root cause of musculoskeletal pain could be infection of the spinal and paraspinal structures, especially in populations that have a complex medical history and are undergoing dialysis. It is possible that spinal infections occur in chiropractic offices. Furthermore, patients with spondylodiscitis may not present with fever, which is a typical presentation. Unusual symptomatology may result in challenging diagnostic and treatment decisions.

Unlike previous cases that presented to the emergency department, this is the first case involving a patient on chronic peritoneal dialysis presenting with spondylodiscitis to a chiropractic clinic. The complex medical history of the patient combined with the unusual presentation of acute-on-chronic back pain underscores the critical role chiropractors can play in the early identification and management of rare and potentially severe conditions in patients with a complicated medical background. Such reports will enrich the knowledge base, inform future practice, and potentially facilitate the earlier recognition of similar cases.

As part of complementary and alternative medicine, chiropractic care plays an important role in the treatment of musculoskeletal symptoms, especially in patients with chronic diseases. Chiropractic care focuses primarily on the diagnosis and treatment of neuromusculoskeletal disorders through manual manipulation or adjustment of the spine [22]. It offers a non-pharmacological approach to pain management that can be particularly beneficial for patients with chronic illnesses who are often already taking multiple medications. In addition, chiropractors often adopt a holistic approach to patient care, considering the patient's lifestyle, diet, exercise, and other habits, which can contribute to overall well-being and quality of life [22]. However, in complex cases, such as in patients on chronic dialysis, chiropractors must be vigilant for signs of serious underlying conditions such as spondylodiscitis and must work closely with other healthcare providers to ensure appropriate and coordinated care [23,24].

In this case, the role of the chiropractor was extended beyond that of the traditional scope of musculoskeletal management. Chiropractors were crucial in recognizing the atypical and severe nature of the patients' symptoms, prompting further investigation and referral. This emphasizes the responsibility of chiropractors in the triage process, which can significantly affect patient outcomes, particularly when dealing with potentially life-threatening conditions such as spondylodiscitis.

Given the complexity of these cases, chiropractors should be encouraged to establish strong professional networks with a wide range of healthcare providers. This can facilitate timely referrals and multidisciplinary case discussions, ensuring comprehensive patient care. Furthermore, continuing professional development and education on the recognition of specific symptoms associated with severe conditions such as spondylodiscitis are crucial to ensure that chiropractors are well-equipped to handle these complex cases.

## Conclusions

This case of spondylodiscitis in a peritoneal dialysis patient presenting to chiropractic care highlights the diagnostic challenges of spinal infections in complex medical cases. It emphasizes the critical triage role of chiropractors in identifying red flags and facilitating specialist referrals when managing patients with comorbidities. This report enriches the literature on an uncommon manifestation of a rare disease. It underscores the need for chiropractors to maintain a high index of suspicion for sinister pathologies like spondylodiscitis in at-risk populations, establish robust referral networks, and continually update their knowledge on recognizing and managing severe conditions that may mimic musculoskeletal disorders. Such vigilance and collaboration are key to ensuring timely diagnosis and optimal patient outcomes.
